# The impact of motivation to lead on team outcomes: the mediating role of leaders’ role satisfaction in China and Germany

**DOI:** 10.3389/fpsyg.2025.1605603

**Published:** 2025-10-20

**Authors:** Stephan Braun, Anna Semenkova, Julia Lalla, Rolf van Dick, Alina S. Hernandez Bark

**Affiliations:** ^1^Department of Psychology, Goethe University Frankfurt, Frankfurt, Germany; ^2^Department of Economics and Law, RH University of Applied Science Cologne, Köln, Germany

**Keywords:** motivation to lead, role satisfaction, team effectiveness, team climate, culture

## Abstract

**Introduction:**

Motivation to lead (MTL) has been identified as a key predictor of leadership effectiveness. It comprises three distinct facets—affective, calculative, and normative MTL—which differentially impact leadership outcomes. However, we know little about how these facets affect team climate and team effectiveness across cultures and from leader and follower perspectives. Additionally, we examine the influence of role satisfaction with the leader role within this relationship.

**Methods:**

We conducted two complementary studies to examine the effects of MTL on team outcomes. Study 1 involved a German leader sample, while Study 2 comprised follower samples from both Germany and China. We measured affective, calculative, and normative MTL as independent variables, team effectiveness and team climate as dependent variables, and additionally role satisfaction with the leader role as a potential mediator.

**Results:**

Our findings confirmed that the three MTL facets have differential effects on team outcomes. Affective MTL consistently showed positive effects across samples. In contrast, calculative and normative MTL demonstrated mixed effects in the different cultural contexts and whether the perspective was a leader or a follower one. Specifically, normative and calculative MTL were perceived more positively in the Chinese follower sample. Mediation analysis revealed that role satisfaction significantly mediated some of the relationship between MTL and outcomes, but only in Germany.

**Discussion:**

These results suggest that research should focus more on boundary conditions of MTL and its effects. Special consideration should be given to the culture in which MTL is measured and who (followers or leaders) provides these assessments. This could inform more nuanced MTL research as well as enable more effective programs in leadership selection and organizational culture development.

## Introduction

Leadership is a phenomenon as old as humankind (Grint, 2011) and its relevance and complexity have only grown in recent years (O'Connell, 2014). Due to automatization, digitalization, new organizational structures, and many other challenges, there is a demand for more leadership and more competent leaders (Bennis and Nanus, 1985). Thus, early identification and further development of employees who have the motivation and potential to become leaders are among the most important tasks of HR departments (Hernez-Broome and Hughes, 2004).

A key possibility for identifying employees with high leadership potential is offered with the Motivation to Lead (MTL) concept by Chan and Drasgow (2001). This motivation theory differentiates between three facets of the willingness to take on a leadership position: (1) Intrinsic interest in leading others (affective MTL), (2) a feeling of being obligated to take on a leading position (normative MTL), and (3) a desire to lead because of additional personal benefits (calculative MTL). Originally, this third component was reverse-coded and labelled non-calculative MTL but other authors (e.g., Felfe et al., 2012) suggested to call and measure it calculative MTL for reasons of simplicity. A recently published meta-analysis (Badura et al., 2020) has shown that affective and normative MTL have positive effects on outcomes of leadership effectiveness, whereas calculative MTL is negatively related to relevant outcomes. Badura et al. (2020) provide a valuable overview of the general effects of different MTL facets, but there are several relevant aspects such as boundary conditions and underlying psychological mechanisms that still have to be researched. Therefore, our main research aim is to find support for a psychological mechanism with which the three MTL facets are differentially affecting the team of the leader and how this is moderated by culture. The psychological mechanism we propose is that the extent to which leaders are satisfied with the social role of being a leader (i.e., their *role satisfaction* with the leader role) mediates the effects of the MTL facets on the team. Taken together, we extend MTL research by making four contributions:

First, we attempt to investigate how the differential effects of MTL facets can be explained. While many studies show that MTL is connected to relevant leadership outcomes (Chan and Drasgow, 2001; Judge and Long, 2012; Kark and van Dijk, 2007; Stiehl et al., 2015), it is still unclear which psychological processes can explain these effects. This study attempts to contribute to this question’s answer. We argue that the leader’s role satisfaction might be a key mediating factor. By adopting the scale of role satisfaction of mothers (Barling and MacEwen, 1988), we capture leader’s role satisfaction as feelings and thoughts about their role as a leader, incorporating their perceptions of the social expectations of being a leader (Schulz-Schaefer, 2018). We argue that the relationship between MTL facets and leadership outcomes can be partly explained via the leader’s satisfaction with their role because how someone is motivated to become a leader should also affect the degree to which he or she is satisfied being in a leader role. This effect is also reflected in positive correlations between MTL and leaders’ job satisfaction (e.g., Maurya and Agarwal, 2018). Role satisfaction, in turn, should affect leaders’ performance, as is the case with job satisfaction (Judge et al., 2001). We use role satisfaction and not job satisfaction because role satisfaction should be conceptually closer to leadership and MTL. After all, it targets the aspect of being a leader directly, leaving out other aspects of the job.

Second, because we want to measure the outcomes of leadership with respect to the team level, we want to include the leader as well as the follower perspectives. The leader’s perspective is relevant because it represents the actual motivation and role satisfaction of the leader. The follower perspective gives us the perception of the leader’s motivation and role satisfaction by a team member. This is also relevant because, for the team, the perceived motivation of a leader should be equally important, as it will influence the image employees have about their leader. Additionally, the follower perspective offers a more proximal measurement of the team outcomes.

Third, this paper aims to examine culture as a relevant factor influencing the effects of the different MTL facets. Especially the interpretation of normative MTL should depend on the importance of these norms and how they are perceived in society, which varies between different cultures (Triandis, 1995). Research on collectivism and individualism implies that it is more important for people from collectivistic societies to follow norms, while the behavior of people from individualistic countries is strongly influenced by personal values (e.g., Saracevic et al., 2022). This should also apply to norms regarding the expectation to become a leader. Therefore, we want to test the impact of normative MTL on relevant outcomes in one individualistic (Germany) and one collectivistic (China) country.

Fourth, we are introducing team-oriented dependent variables, precisely team-effectiveness and team climate, into the MTL research. So far, studies in this field have almost exclusively focused on outcomes of MTL regarding the leader, like leadership emergence, leadership styles, and leadership effectiveness (e.g., Hong et al., 2011; Badura et al., 2020). Yet, if one understands leadership as enhancing team performance, team-oriented variables have to be included in this research as well. While there are individual findings regarding the effects of MTL on individual followers (Auvinen et al., 2020) or team performance (Hendricks and Payne, 2007), to the best of our knowledge, effects of MTL on the team beyond pure performance have not been studied so far.

**Figure 1:**
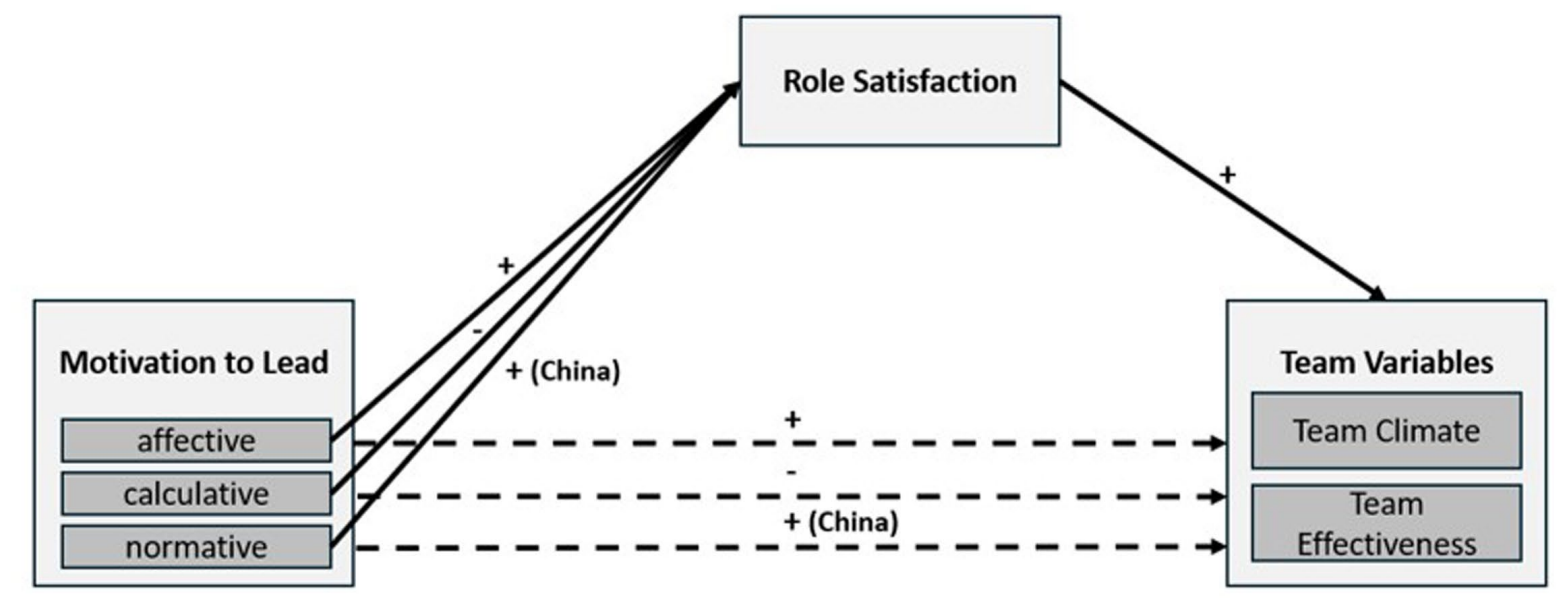
Proposed research model.

Thus, we propose this research model (see Figure 1) which we examine in both China and Germany and from both follower and leader perspective. In the following, we first provide the theoretical development of our hypotheses constituting our research model. Second, we present and describe the methods and results of both, the German and Chinese, studies. Third, we summarize the main findings, discuss their theoretical and practical implications and outline prospects for future research.

### Motivation to lead

After a long period of research focusing on employee motivation and its positive effects, with a focus on how managers influence this through their behavior, in recent years research also focuses on leader motivation itself (Elprana and Felfe, 2019). As one of the first approaches to examine leader motivation, the motivation to lead (MTL) construct was introduced to provide a possible explanation for the occurrence of different leadership behaviors within this framework (Chan and Drasgow, 2001). Accordingly, MTL forms a construct of individual differences that describes the extent and reason of the will to assume leadership roles and leadership responsibilities as well as the intensity of leadership effort.

Thus, it differentiates between three different motivation to lead facets, namely affective, (non-)calculative, and (social-)normative MTL. (1) Affective MTL encompasses a motivation to lead because of the enjoyment and pleasure felt in the activity itself. (2) Non-calculative MTL describes the extent to which a person assumes leadership without weighing the costs and potential gains, i.e., assuming leadership regardless of one’s own benefits or losses. As mentioned above, we follow Felfe et al. (2012) who label this facet calculative MTL, the motivation for leadership because of its benefits. (3) Normative MTL describes the motivation to lead based on a sense of obligation and responsibility. Here, assuming a leadership position is driven by the expectations of the social or societal environment. This three-component model of MTL was confirmed by numerous studies (for an overview see the meta-analysis by Badura et al., 2020).

Studies on MTL have also demonstrated its relevance in terms of leadership outcomes. Chan and Drasgow (2001) showed in their validation study on MTL that leadership motivation was associated with behavioral measures of leadership potential. Auvinen et al. (2020) used profile analyses to show that leader’s MTL structure affects multiple different outcomes like career intentions, occupational health, well-being, work engagement, burnout, and Leader-Member-Exchange. The recent meta-analysis by Badura et al. (2020) examined the differential effects of the three MTL facets on leadership emergence, leadership styles, and leader effectiveness. Here, affective leadership motivation proved to be the strongest predictor for leadership emergence and leadership effectiveness. Normative MTL was the strongest predictor for the use of transactional leadership, a leadership style characterized by the use of reward and punishment and by appealing to the self-interest of the follower (Odumeru and Ogbonna, 2013). Calculative MTL was negatively connected with effective leadership styles like transformational leadership and positively related with destructive leadership styles such as laissez-faire leadership.

To explain the effects of different sources of motivation (as is the case for the MTL), the Self-Determination Theory (SDT) by Deci and Ryan (1985) provides a relevant theoretical foundation. SDT bases motivation on different types of incentive sources, distinguishing between intrinsic and extrinsic motivation. Intrinsic motivation is thereby attributed to a purely internal interest of the person, independent of external incentives. Intrinsically motivated individuals perform activities because they find these activities interesting and enjoyable. In contrast, extrinsic motivation describes behavioral impulses aimed at an outcome that is separable from the activity, for instance financial rewards (Ryan and Deci, 2017). Research has shown repeatedly that intrinsic motivation is connected to better work performance (e.g., the meta-analysis by Cerasoli et al., 2014). In contrast, the effect on extrinsic motivation like financial incentives on performance is not as consistent. It can have positive effects (e.g., Makki and Abid, 2017), no effect or even negative effects (e.g., Kuvaas et al., 2017) on performance. According to the SDT, this depends on whether the external motivation is interpreted positively, as something that adds value to a self-selected goal or negatively, as an external means of control and regulation (Gerhart and Fang, 2015; Gagné and Deci, 2005). Kanat-Maymon et al. (2020) have applied this mechanism to leadership research. They showed that intrinsic and extrinsic work motivation of leaders are differentially predicting leadership styles of the full range model of leadership (Bass, 1996), and these leadership styles again are influencing followers’ motivation. What is missing in this line of research so far is the introduction of actual leadership motivation. Kanat-Maymon et al. (2020) are using a work motivation scale which not explicitly incorporates the role and the responsibilities of the leader role. Thus, to our knowledge, we are the first to theoretically connect the two important lines of motivation research SDT and MTL.

Using SDT to categorize the MTL facets, calculative and normative leadership motivation are both different kinds of extrinsic motivation that should be usually perceived as high in external control. Whereas calculative leadership motivation is aimed at an individual benefit such as better pay, to motivate taking a position that the person otherwise would not have pursued, normative leadership motivation is driven by the expected reactions of the social environment. Thus, normatively motivated individuals want to fulfill the expectations of their social environment and, for example, avoid negative reactions that could occur if they do not take on the leadership position, they are offered. This could explain Badura et al.’s (2020) finding that normative MTL is the best predictor for transactional leadership because this is a leadership style that is also based on extrinsic motivation. In contrast, affective leadership motivation is the only one of the three facets that reflects intrinsic motivation and thus an internal incentive through which the leadership activity itself is perceived as a reward (Furtner and Baldegger, 2016).

### MTL and its effects on the team

Research on leadership identified a wide range of different outcomes and perspectives to measure the effects of leadership. These outcomes can focus on the leader, the follower, the team, or the organization and can measure amongst other things effectiveness, attitudes, behavior, or cognition (for an overview see Hiller et al., 2011). So far, MTL research has mainly focused on leader-oriented measures. For our research, we wanted to widen the scope of MTL research by introducing team-oriented measures that can be assessed by leaders as well as by followers. Therefore, we chose team effectiveness and team climate as two relevant dependent measures. These indicators are inferred from the main leadership tasks: leading a team to the achievement of the organizational goals (team performance) and managing processes within the team (team climate) (Nerdinger, 2019b; Yukl, 2010).

Team effectiveness can be defined as the extent to which given goals have been achieved (Ramírez-Mora et al., 2019). Previous research in various cultures has shown that leadership is an important factor in team outcomes (e.g., Durham et al., 1997; Niu et al., 2024; Rahmadani et al., 2020; Santos et al., 2015). However, most of these studies focused on the effects that leaders’ behavior or leadership style can have on team outcomes whereas the effects of leader motivation remain understudied. To the best of our knowledge, there is only one study that investigated the effects of MTL on team effectiveness. Hendricks and Payne (2007) conducted an experiment in which they examined the effects of MTL on perceived leadership effectiveness and team performance. Positive, marginally significant effects on objective team performance were found only for affective MTL which was also marginally positively connected to leadership effectiveness while both calculative and normative MTL had negative effects.

Because not only the achievement of goals but also processes within the team are an important outcome of leadership (Nerdinger, 2019a), we chose team climate as a second indicator for effective leadership. Team climate can be defined as the individual or shared perception of the situation in the team (Brodbeck et al., 2000). It can be studied as a whole construct or, more commonly, researchers focus only on specific aspects. We chose two such aspects, namely participative safety and task orientation. Participative safety means that team members experience their work environment as safe, motivating them to participate actively in all team processes (Brodbeck et al., 2000). Task orientation refers to team member behavior that leads to high-quality performance. It is often characterized by regular evaluation and feedback by team members (Thayer et al., 2018). We decided on these aspects of team climate because, on the one hand, they lead to the achievement of organizational goals and, on the other hand, they involve team members’ well-being. So far, we do not know of any research on the relationship between MTL and team climate. However, many studies showed that leaders’ behavior and their leading style can affect team climate (e.g., Gonzalez-Roma et al., 2002; Kinnunen et al., 2016; Woolley et al., 2011; Yang et al., 2019; Zohar and Luria, 2004; Zohar and Tenne-Gazit, 2008). In addition, leader personality traits (e.g., Big-Five) can influence some aspects of team climate (Mayer et al., 2007). Therefore, it can be assumed that MTL might affect team climate, too.

### The influence of culture on MTL

Norms and the consequences of (not) conforming to them depend strongly on national culture (Mc Breen et al., 2011). Therefore, culture should particularly influence the perception of normative MTL. To date, there is no research on how culture affects outcomes of MTL. However, there is some first evidence of effects of MTL facets on culture-related constructs showing that vertical collectivism is a strong predictor of normative and (non-)calculative MTL (Badura et al., 2020; Oh, 2012).

It is generally believed that in collectivist cultures, the common good is prioritized over the individual’s interests, which is also true for leaders in China (Campion and Wang, 2019; Yang et al., 2008). Since these interests are deemed morally important by society, it can be hypothesized that normative leadership motivation is also viewed more positively in Eastern societies than in Western societies. Few studies have examined MTL in China, but in a study by Chen (2016), two significant positive correlations were found between positive leader identity and affective and normative MTL, respectively. Whether there are actual cultural differences in the effects of MTL on relevant outcomes has not been researched so far.

For the predictions of our studies which focus on the effects of MTL on team-related outcomes, we consider prior findings regarding the effects of the MTL facets as well as the logic of the SDT and an effect of culture. For affective MTL we predict consistent positive results, because of the consistent positive effects in prior research on outcomes and positive leadership styles as well as its intrinsic nature. In contrast, for calculative MTL we predict consistent negative results because of its consistent negative effects in prior research on outcomes, its connection to negative leadership styles and its extrinsic nature. For normative MTL we predict a more differentiated picture. On the one hand, the meta-analysis by Badura et al. (2020) showed a weak positive effect on leadership effectiveness and positive correlations with transformational leadership as well as transactional leadership. On the other hand, normative MTL is an extrinsic motivation and Hendricks and Payne (2007) found no effects on team performance (which is especially relevant for this study) and negative effects on perceived leadership effectiveness. Because of this mixed evidence, we predict positive effects of normative MTL on team-related outcomes for the Chinese sample only, because normative motivation – despite being more extrinsic - should be valued positively due to the collectivistic culture (Hofstede, 1984) and interdependent construction of the self (Markus and Kitayama, 1991) in China. In contrast, there is no consistent evidence for either a positive or negative relation for Germany. Also, we do not make any differential predictions based on the perspective (leader or follower), as we assume the relations to be equivalent regarding their valence for both.

Thus, our first two sets of hypotheses are:

*Hypothesis 1a: Affective MTL correlates positively with higher team effectiveness.*

*Hypothesis 1b: Calculative MTL correlates negatively with team effectiveness.*

*Hypothesis 1c: Normative MTL correlates positively with team effectiveness – but only in the Chinese sample.*

*Hypothesis 2a: Affective MTL correlates positively with team climate.*

*Hypothesis 2b: Calculative MTL negatively correlates with team climate.*

*Hypothesis 2c: Normative MTL correlates positively with team climate – but only in the Chinese sample.*

### Motivation to lead and role satisfaction

Research interest in employee satisfaction has existed for many years and is based on the finding that job satisfaction is associated with different economic outcomes (Ferreira, 2019). Many studies showed that job satisfaction is positively related to performance (Fischer and Fischer, 2005; Judge et al., 2001; Schmidt et al., 2007) and well-being (Ilies et al., 2015; Karabati et al., 2019; Schmidt et al., 2007), and negatively related to turnover and internal resignation (Acikgoz et al., 2016; Gebert and von Rosenstiel, 2002). Job satisfaction can be seen as a general construct consisting of different facets (Judge et al., 2017). In our study, we introduce a new facet of job satisfaction: role satisfaction. A role is a sum of expectations about behavior and responsibilities of a person holding a certain position (Schulz-Schaefer, 2018). Thus, role satisfaction is an emotional state that a person experiences when thinking about one’s role and the associated experiences (Barling and MacEwen, 1988). To date, research on role satisfaction has been limited primarily to family–work conflict or parental role satisfaction. Studies in the area showed that role perception and role satisfaction can influence people’s well-being and health (Baruch and Barnett, 1986; Martire et al., 1997; Wickrama et al., 1995). However, role satisfaction has not been introduced to the leadership context so far. With this study, we aim to illustrate the importance of this construct for the work context. Particularly, we assume that the relationship between the different MTL facets and team effectiveness or team climate, respectively, can be mediated by the leaders’ satisfaction with their role as a leader.

The relationship between motivation and satisfaction is a controversial topic in research. For example, von Rosenstiel et al. (2005) describe the relationship between motivation and satisfaction in the following way: Internal motives generate certain needs, the fulfillment of which leads to satisfaction. Thus, it can be assumed that individuals who have sought a leadership position because of an intrinsic interest in leadership tasks, are more satisfied with the possibility to perform leaders´ tasks and their leaders’ role. This can also be explained by the model of Herzberg et al. (1959). They identified two groups of work factors: hygiene factors and motivators. The motivators lead to positive work attitudes, which in turn can increase job satisfaction. Their absence, however, does not lead to dissatisfaction, but to a neutral state, in other words, a zero-satisfaction. The absence of hygiene factors, on the other hand, leads to dissatisfaction with the job. However, the hygiene factors cannot increase satisfaction. Interestingly, motivators correspond to intrinsic motivation and hygiene factors to extrinsic motivation (Tietjen and Myers, 1998). Therefore, only intrinsic motivation can increase satisfaction. Based on this, we assume that only affective MTL as the intrinsic facet of MTL can positively influence role satisfaction, but not the two extrinsic facets of MTL (calculative and normative), except normative MTL in China, because of the already mentioned cultural differences in the perception of normative motivation. Therefore, our next hypotheses are:

*Hypothesis 3a: Affective MTL correlates positively with role satisfaction.*

*Hypothesis 3b: Calculative MTL correlates negatively with role satisfaction.*

*Hypothesis 3c: Normative MTL correlates positively with role satisfaction – but only in the Chinese sample.*

Using the concept of role satisfaction, we are aiming to give a possible explanation of why high affective MTL leads to better leadership effectiveness. We hypothesize that role satisfaction plays a mediating role in the relationship between MTL and leadership effectiveness, such that leaders with high intrinsic (i.e., affective) MTL are more satisfied with their leadership roles. More satisfied leaders in turn should perform their leadership tasks more effectively. Judge et al. (2001) critically analyzed previous studies and meta-analyses on the connection between satisfaction and performance of leaders and they report an average correlation of 0.30 between both concepts. The studies on organizational or group levels also show significant correlations between job satisfaction and various job performance criteria, ranging from 0.20 to 0.31 (Ostroff, 1992; Ryan et al., 1996; Schmidt et al., 2007). Considering these findings, we assume that role satisfaction also correlates positively with leadership performance, represented in our study by team performance and team climate, and can therefore mediate the relationship between MTL and team effectiveness or team climate, respectively.

This results in the following set of hypotheses:

*Hypothesis 4a: Role satisfaction has a mediating role between affective MTL and team effectiveness: The higher affective MTL, the higher the role satisfaction, and higher role satisfaction leads to higher team effectiveness.*

*Hypothesis 4b: Role satisfaction has a mediating role between affective MTL and team climate: The higher affective MTL, the higher the role satisfaction, and higher role satisfaction leads to a better team climate.*

*Hypothesis 4c: Role satisfaction has a mediating role between calculative MTL and team effectiveness: The lower calculative MTL, the higher the role satisfaction, and higher role satisfaction leads to higher team effectiveness.*

*Hypothesis 4d: Role satisfaction has a mediating role between calculative MTL and team climate: The lower the calculative MTL, the higher the role satisfaction, and higher role satisfaction leads to a better team climate.*

*Hypothesis 4e: Role satisfaction has a mediating role between normative MTL and team effectiveness: The higher normative MTL, the higher the role satisfaction, and higher role satisfaction leads to higher team effectiveness – but only in the Chinese sample.*

*Hypothesis 4f: Role satisfaction has a mediating role between normative MTL and team climate: The higher normative MTL, the higher the role satisfaction, and higher role satisfaction leads to a better team climate – but only in the Chinese sample.*

## Study 1

### Methods

#### Procedure

We used the service QuestBack (unipark.de) to create an online questionnaire for the first study. The questionnaire was in German. The English scales (role satisfaction, team climate, and team effectiveness) were translated into German by two bilingual researchers with one translating the scales to English and the other one doing a back translation to German (following the approach by Brislin, 1986). All scales were constructed in the form of a self-report.

Participants were recruited on the professional networking platform XING as well as via personal networks. The participants were informed that the participation is voluntary, anonymous, and without any profits. Only participants with leadership responsibility who fully completed the questionnaire were included in the analyses.

#### Sample

One hundred thirty participants completed the questionnaire to the end. One person had to be excluded from the study due to not having any leadership responsibility. Therefore, the data of 129 participants (92 male, 37 female) were analyzed. Participants’ age ranged from 23 to 61 years (*M* = 41.91, *SD* = 8.7) and their work experience ranged from 4 to 42 years (*M* = 19.67, *SD* = 9.58), of which 1 to 35 years came with leadership experience (*M* = 10.82, *SD* = 8.11). They were responsible for between 1 and 4,200 employees (*M* = 119.88, *SD* = 459.94), of whom 1 to 50 employees reported directly to the participant (*M* = 7.74, *SD* = 7.56). Hierarchical level was distributed as follows: 16.3% operational management, 55% middle management (department, division, or area management), and 28.7% top management (strategic management of an organization). Participants were employed in a variety of industries: 18% from consulting, 13% from education and research, 12% from industry, 11% from traffic and transportation, 9% from services, 8% from IT, 6% from finance, 3% from real estate, 2% from non-profit organizations, and 17% from other industries.

#### Instruments

##### Motivation to lead

To measure the three facets of MTL, we used the scales from the Hamburger MTL inventory (Hamburger Führungsmotivationsinventar, FÜMO, Felfe et al., 2012). To shorten the participation time, we excluded repetitive and similar items as well as items with low discriminatory power. In the final version, we used six items for affective MTL (*α* = 0.67). Example item: “I like to take responsibility for others.” We used four items for Calculative MTL (α = 0.75), example item:” I am only interested in leading a group if there are clear advantages for me.” And we used four items for normative MTL (α = 0.75). Example item: “I feel obliged to take the lead when I am asked to do so.” All items were measured in the format of a 5-point Likert scale (from 1 – “strongly disagree” to 5 – “strongly agree”). An explorative factor analysis and a Varimax Rotation confirmed that the selected items represent the corresponding facets of MTL.

##### Team effectiveness

The team effectiveness scale was adapted from the team effectiveness scale developed by Ramírez-Mora et al. (2019). Five of the seven items were chosen, translated into German, and adapted to the managers’ perspective (α = 0.82). Example item: “As a team, we achieve our goals.” Two items had to be excluded because they were industry specific and could not be applied to all participants. The answers scored from 1 – “strongly disagree” to 5 – “strongly agree.”

##### Team climate

Team climate was measured with seven items (α = 0.87). For this, the “participative safety” and “task orientation” scales from Strating and Nieboer (2009) were used. Example item: “The team members feel mutually accepted and understood.” The answers scored from 1 – “strongly disagree” to 5 – “strongly agree.”

##### Role satisfaction

The role satisfaction scale was adapted from Barling and MacEwen (1988). The seven items were translated into German and adapted to the managers’ role (α = 0.80). Example item: “Overall, I am very satisfied with my role as a manager.” The answers scored from 1 – “strongly disagree” to 5 – “strongly agree.”

### Results

The data were analyzed using the IBM SPSS program. The means, standard deviations, reliabilities and correlations of all variables used in the first study are presented in Table 1. Previous studies have shown that there is a gender difference in MTL with women showing lower affective and normative MTL (Hernandez Bark et al., 2016; Elprana et al., 2015; Schuh et al., 2014). However, including gender in our mediation model had no relevant influence on the results, therefore we did not include it in our final analyses.

**Table 1:** Means, standard deviations, reliabilities, and correlations for the German leader sample.

Variables	*M*	*SD*	1	2	3	4	5	6	7	8
1. Gender	1.29	0.45	–							
2. Age	41.91	8.70	0.01	–						
3. Affective MTL	4.01	0.51	−0.19*	−0.06	(0.67)					
4. Calculative MTL	2.18	0.75	0.03	−0.14	−0.11	(0.75)				
5. Normative MTL	2.70	0.83	−0.23**	−0.04	0.08	−0.01	(0.75)			
6. Role satisfaction	4.18	0.50	−0.06	−0.05	0.46***	−0.25**	0.04	(0.80)		
7. Team effectiveness	3.92	0.57	0.05	0.09	0.16^†^	−0.13	−0.16^†^	0.40***	(0.82)	
8. Team climate	4.00	0.60	0.11	−0.08	0.23**	−0.06	−0.14	0.35***	0.54***	(0.87)

*N* = 129. Gender: 1 = male, 2 = female; Cronbach’s alphas in parentheses; MTL = motivation to lead. ***p < 0.001; **p < 0.01 (two-sided); *p < 0.05 (two-sided); ^†^*p* < 0.10 (two-sided).

The three facets of leadership motivation correlated positively with general leadership motivation, but did not significantly correlate with each other, confirming the three-dimensional structure of MTL suggested by Chan and Drasgow (2001).

Confirming Hypothesis 1a, we found a positive, marginally significant correlation between team effectiveness and affective MTL (*r* = 0.16, *p* = 0.07). Contrary to our expectations, no significant correlation between team effectiveness and calculative MTL and a marginally significant negative correlation between team effectiveness and normative MTL (*r* = −0.16, *p* = 0.08) were found. Thus, Hypothesis 1a could be confirmed and Hypothesis 1b was rejected (see Table 1). We also did not predict a negative effect of normative MTL on team effectiveness.

Similar to the first set of hypotheses, we assumed in our second set a positive correlation between affective MTL and team climate (Hypothesis 2a), as well as negative correlation between team climate and calculative MTL (Hypothesis 2b). Hypothesis 2a was confirmed whereas 2b was not. We found a positive significant correlation between team climate and affective MTL (*r* = 0.23, *p* < 0.05), and no correlations between team climate and the two other facets of MTL (see Table 1).

Our third set of hypotheses suggested again a positive correlation between affective MTL (Hypothesis 3a) and role satisfaction and a negative correlation between calculative MTL and role satisfaction (Hypothesis 3b). Here, both hypotheses were confirmed. We found a positive correlation between affective MTL and role satisfaction (*r* = 0.46, *p* < 0.001), a significant negative correlation between calculative MTL and role satisfaction (*r* = −0.25, *p* < 0.01). Normative MTL and role satisfaction did not correlate (see Table 1).

#### Mediation analysis

To test our further hypotheses, we calculated the mediation effects of role satisfaction on the relationship between different facets of MTL and team effectiveness or team climate, respectively. For this, we used the Model 4 of Macros PROCESS (Hayes, 2018) in IBM SPSS. Regarding the calculation of the mediation models, we follow Zhao et al. (2010) and Rucker et al. (2011) who argue that a significant total effect is not a prerequisite for a mediation effect. Instead, a significant indirect effect (Rucker et al., 2011; Zhao et al., 2010) is crucial for the existence of a mediation effect. This means that the mediation analysis can also be calculated for models without a significant total effect.

With our fourth hypotheses set, we suggested a positive mediation effect of role satisfaction on the relationship between affective MTL and team effectiveness (Hypothesis 4a) as well as team climate (Hypothesis 4b) and a negative mediation effect of role satisfaction on the relationship between calculative MTL and team effectiveness (Hypothesis 4c) as well as team climate (Hypothesis 4d). Results are presented in Table 2.

**Table 2:** Mediation effects of role satisfaction between MTL and team effectiveness and team climate in the German leader sample.

Variables and indices	Role satisfaction		Team effectiveness		Team climate	
	*b*	*SE*	*b*	*SE*	*b*	*SE*
Affective MTL	0.45***	0.08	−0.03	0.10	0.10	0.06
Role Satisfaction	--------	--------	0.47***	0.10	0.37**	0.11
*R^2^*	0.21		0.16		0.13	
Total, direct and indirect effect	--------		Total effect: 0.18^†^; direct effect: −0.03; indirect effect: 0.21, *SE* = 0.07 (*CI*95 0.09, 0.37)		Total effect: 0.27**; direct effect: 0.10.; indirect effect: 0.17, *SE* = 0.06 (*CI*95 0.07, 0.29)	
Calculative MTL	−0.17**	0.06	−0.03	0.06	0.03	0.07
Role Satisfaction	--------	--------	0.45***	0.10	0.43***	0.10
*R^2^*	0.06		0.16		0.13	
Total, direct and indirect effect	--------		Total effect: −0.10; direct effect: −0.03; indirect effect: −0.07, *SE* = 0.03 (*CI*95–0.15, −0.02)		Total effect: −0.05; direct effect: 03.; indirect effect: −0.07, *SE* = 0.3 (*CI*95–0.14, −0.03)	

*N* = 129. MTL = motivation to lead, Bott *SE* = standard error of indirect effect, LL = lower limit, UL = upper limit, corr. 95% *CI* = corrected 95% confidence interval, 5,000 bootstrap replicates; ***p < 0.001; **p < 0.01 (two-sided); ^†^*p* < 0.10 (two-sided).

**Figure 2:**
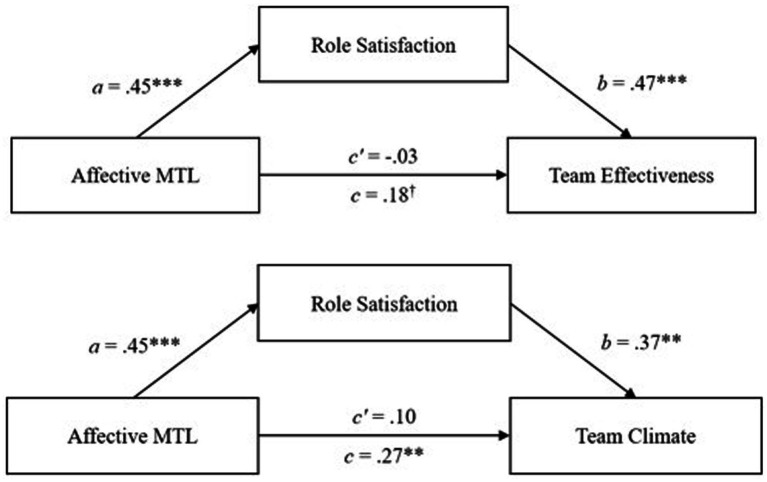
The mediation effects of role satisfaction on the relationship between affective MTL and team outcomes in the German leader sample. ***p < 0.001, ** < 0.01, ^†^*p* < 0.10.

The indirect effect of role satisfaction on the relationships between affective MTL and team effectiveness was 0.21, 95% *CI* [0.09; 0.37] with a positive relationship with affective leadership motivation (*a* = 0.45, *p* < 0.001) and with team effectiveness (*b* = 0.47, *p* < 0.001) (see Figure 2). On the relationships between affective MTL and team climate, role satisfaction had an indirect effect of 0.17, 95% *CI* [0.07; 0.29] with a positive relationship with affective leadership motivation (*a* = 0.45, *p* < 0.001) and with team climate (*b* = 0.37, *p* < 0.01) (see Figure 2). Both indirect effects were positive and significant, confirming our Hypotheses 4a and 4b. As predicted, role satisfaction plays a mediating role between affective MTL and team effectiveness and team climate, respectively. All paths were positive, so higher affective MTL led to higher role satisfaction, and higher role satisfaction in turn had positive effects on team effectiveness and team climate, respectively.

**Figure 3:**
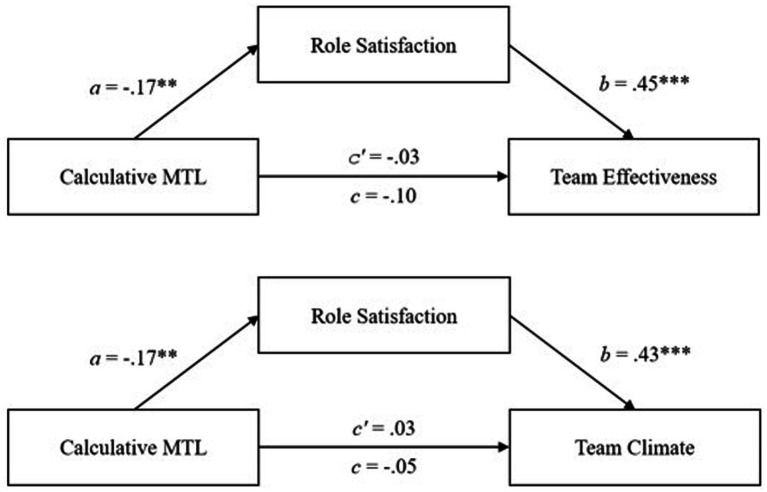
The mediation effects of role satisfaction on the relationship between calculative MTL and team outcomes in the German leader sample. ***p < 0.001, ** < 0.01.

A significant negative indirect effect of role satisfaction on the relationships between calculative MTL and team effectiveness (−0.07, 95% *CI* [−0.15; −0.02]) with a negative relationship with calculative leadership motivation (*a* = −0.17, *p* < 0.01) and a positive path to team effectiveness (*b* = 0.45, *p* < 0.001) (see Figure 3) were found. Also, a significant negative indirect effect of −0.07, 95% *CI* [−0.14; −0.03] was found for the relationships between calculative MTL and team climate. The path between role satisfaction and calculative MTL was *a* = −0.17 (*p* < 0.01) and the path between role satisfaction and team climate was *b* = 0.43 (*p* < 0.001) (see Figure 3). Again, in these mediation analyses, the direct effects of calculative MTL on team effectiveness or team climate were not significant. This means that there is a complete mediation of these effects by role satisfaction. These effects indicated that high calculative MTL leads to lower role satisfaction, which in turn negatively affects team effectiveness or team climate.

## Study 2

### Method

#### Procedure

As in the first study, QuestBack (unipark.de) was used to create two online questionnaires, one for the German and one for the Chinese sample. For the German sample, we used the same scales as in the first study. The wording was changed from the first-person perspective to the third-person perspective for employees to answer the items for their direct leader. For the Chinese sample, we translated all scales into Chinese (Mandarin). This was done by a Chinese native speaker and controlled by a researcher on German-Chinese didactics, again following Brislin (1986). The questionnaire was distributed through personal networks, namely on Facebook, WeChat, LinkedIn, and the SurveyCircle platform.

#### Sample

The German employee sample consists of *N* = 102 individuals (68 female and 34 male). Participants were between 20 and 65 years old with a mean age of *M* = 31.35 (*SD* = 12.16). On average, they had *M* = 9.56 years of work experience (*SD* = 10.86), with a minimum of zero years and a maximum of 40 years. Employees had worked with their current leader up to 15 years (*M* = 2.46, *SD* = 2.84) with a mean team size of *M* = 16.05 people (*SD* = 19.58). 86.54% of the employees did not hold a management position, 6.73% reported working in lower management (operational management) and another 6.73% in middle management (department, division, or division management). Employee estimates show that 38.46% of their managers have over 20 years of professional experience, followed closely by 36.54% with between 11 and 20 years of professional experience. 25% of managers are from lower management, 39.42% are from middle management, and 35.58% are from upper management (strategic management). A majority of 47.12% of the leaders held their position for 1–5 years with a majority of five to 20 employees under their leadership (55.77%). The participants had various backgrounds, most participants came from the service industry (25%), followed by education and research (14.42%), consulting (8.65%) and, traffic and transportation (8.65%).

For the Chinese employee sample, data was collected from 165 individuals. One person was removed from the data analysis because that person did not work in a team, leaving 164 participants. The age of the participants ranged from 19 to 57 years with a mean age of *M* = 30.65 years (*SD* = 8.05). One hundred eleven subjects were female, 52 were male, and 1 was diverse. Employees reported 0 to 38 years of work experience (*M* = 7.71, *SD* = 8.07) of which they worked under their current manager for an average of *M* = 3.16 years (*SD* = 4.01). An average team contained *M* = 18.26 people (*SD* = 24.78). 53.66% of the employees did not hold a management position. 28.05% worked in lower management, 15.85% in middle management, and 2.44% in upper management. The direct manager of the participants was mostly between 31 and 40 years old and 59.15% were male. 40.85% were female. The most frequent estimated duration of work experience of the direct manager was 11 to 20 years (36.59%), followed by the 5 to 10 years category (27.44%). By a significant margin, most managers worked in middle management (50.61%), followed by senior management at 29.88%, and lastly, lower management at 19.51%. In their current position as direct managers, half (49.39%) had been between 1 to 5 years, with mostly 5 to 20 employees under their responsibility (47.56%). The most common industry category was 17.68% from education and research or other industries, respectively. 16.36% of the participants came from the IT industry, 11.59% from the industry, 10.37% from finance, 9.76% from the service industry and 8.54% from consulting.

#### Instruments

The same items and scales as in the first study were used with the difference that employees were asked to evaluate their current leader’s MTL and role satisfaction as well as the effectiveness and the climate of their team.

### Results

R Studio version 1.1.463 was used to calculate the data analyses. As in the first study, Model 4 of the PROCESS macro by Hayes (2018) was used here for the mediation analysis.

#### Results for the German sample

For the German sample, the same hypotheses as in the first study were assumed. Table 3 depicts the means, standard deviations, reliabilities and intercorrelations of the measured scales. In contrast to the other samples, two of the scales (affective MTL and normative MTL) had less than satisfactory reliabilities with Cronbach’s Alphas of 0.60 for affective MTL and 0.59 for normative MTL. This might be due to a somewhat smaller sample and the fact that we used shortened scales.

**Table 3:** Means, standard deviations, reliabilities, and correlations of the German employee sample.

Variables	*M*	*SD*	1	2	3	4	5	6	7	8	9	10
1. Gender Participant	1.67	0.47	–									
2. Age participant	31.35	12.16	0.24*	–								
3. Gender leader	1.43	0.50	0.20*	0.06	–							
4. Age leader	2.78	1.0	0.12	0.35***	0.01	–						
5. Affective MTL	3.49	0.60	0.08	−0.10	0.04	−0.07	(0.60)					
6. Calculative MTL	2.32	0.86	0.02	0.19^†^	−0.03	0.24*	0.06	(0.85)				
7. Normative MTL	3.16	0.67	0.08	0.04	−0.25*	−0.15	0.19^†^	0.04	(0.59)			
8. Role satisfaction	3.93	0.60	0.13	−0.19^†^	−0.03	−0.22*	0.47**	−0.30**	0.04	(0.83)		
9. Team effectiveness	3.98	0.57	0.12	−0.22*	−0.09	−0.22*	0.19^†^	−0.45***	−0.08	0.46***	(0.86)	
10. Team climate	3.98	0.61	0.11	−0.19^†^	−0.08	−0.27**	0.09	−0.46***	−0.08	0.32**	0.65***	(0.87)

*N* = 102. Gender: 1 = male, 2 = female; Cronbach’s alphas in parentheses; Age Leader: 1 = under 30; 2 = 31–40; 3 = 41–50; 4 = 51–60; 5 = over 60; MTL = motivation to lead; ***p < 0.001; **p < 0.01 (two-sided); *p < 0.05 (two-sided); ^†^*p* < 0.10 (two-sided).

We again controlled for gender but as it did not have any effect on the results, we report results without controls. In contrast to the leadership sample, where no inter-correlations between the MTL facet measures were found, in this sample we found a marginally significant positive correlation between affective and normative MTL of 0.19 (*p* = 0.06).

Regarding the first hypotheses set, affective MTL showed a marginally significant positive correlation with team effectiveness (*r* = 0.19, *p* = 0.06) and a negative correlation was found for calculative MTL (*r* = −0.47, *p* < 0.001). Normative MTL did not significantly correlate with team effectiveness. Thus, Hypotheses 1a and 1b were confirmed (see Table 3).

For the second set of hypotheses, regarding team climate, no significant effect was found for affective MTL, a negative correlation for calculative MTL (*r* = −0.46, *p* < 0.001) and no correlation was found for normative MTL. Therefore, Hypothesis 2b was confirmed, while 2a was rejected (see Table 3).

For the third set of hypotheses, affective MTL showed a positive correlation with role satisfaction (*r* = 0.47, *p* < 0.001). A negative correlation was found for calculative MTL (*r* = −0.30, *p* < 0.01) and a non-significant correlation for normative MTL. Thus, both hypotheses were confirmed (see Table 3).

#### Mediation analysis in the German sample

**Figure 4:**
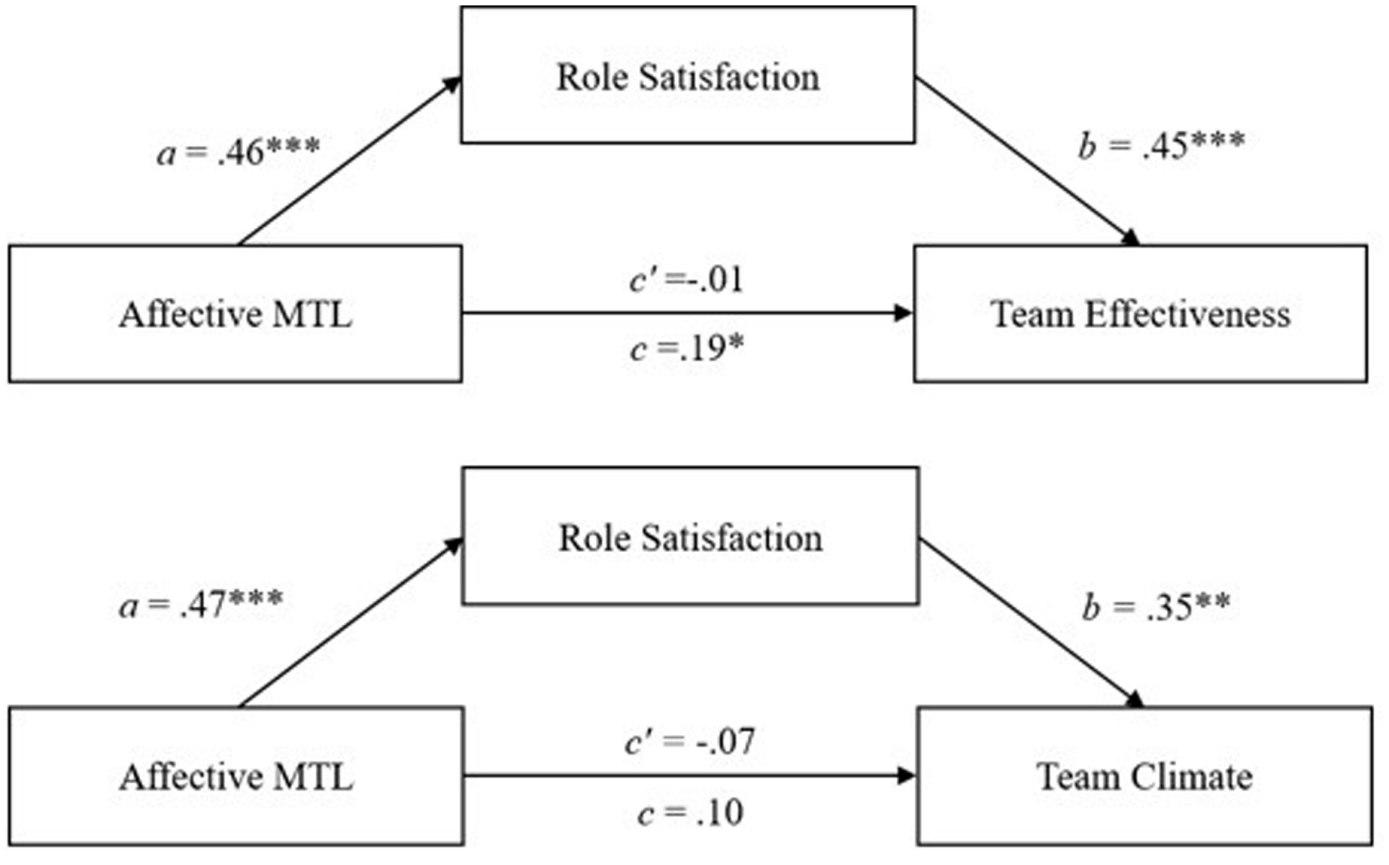
The mediation effects of role satisfaction on the relationship between affective MTL and team outcomes in the German follower sample. ***p < 0.001, ** < 0.01, *p < 0.05.

The mediation analysis (see Table 4) found an indirect effect on the relationship between affective MTL and team effectiveness mediated by role satisfaction (0.20, 95% *CI* [0.08; 0.36]). The total effect of affective MTL on team effectiveness is significantly positive (*c* = 0.18, *p* = 0.048), whereas the direct effect of affective MTL on team effectiveness is not significant (*c′* = −0.01) (see Figure 4). Therefore, we can speak of a complete mediation of role satisfaction on the relationship between affective MTL and team effectiveness. For the relationship between affective MTL and team climate-mediated by role satisfaction, total effect (*c* = 0.10) and the direct effect (*c′* = −0.07) are both not significant. The indirect effect was significant (0.17, 95% *CI* [0.04; 0.33]; see Figure 4). Hypotheses 4a and 4b were thus confirmed.

**Table 4:** Mediation effects of role satisfaction between MTL and team effectiveness and team climate in the German employee sample.

Variables and indices	Role satisfaction		Team effectiveness		Team climate	
	*b*	*SE*	*b*	*SE*	*b*	*SE*
Affective MTL	0.46***	0.09	−0.01	0.10	−0.07	0.10
Role satisfaction	--------	--------	0.45***	0.10	0.35**	0.11
*R^2^*	21.		0.21		0.10	
Total, direct and indirect effect	--------		Total effect: 0.18*; direct effect: −0.01; indirect effect: 0.20, *SE* = 0.07 (*CI*95 0.08, 0.36)		Total effect: 0.10; direct effect: −0.07.; indirect effect: 0.17, *SE* = 0.07 (*CI*95 0.04, 0.33)	
Calculative MTL	−0.21**	0.07	−0.21***	0.06	−0.29***	0.07
Role satisfaction	--------	--------	0.34***	0.08	0.20*	0.09
*R^2^*	0.09		0.30		0.24	
Total, direct and indirect effect	--------		Total effect: −0.28***; direct effect: −0.21***; indirect effect: −0.07, *SE* = 0.04 (*CI*95–0.15, −0.01)		Total effect: −0.33***; direct effect: −0.29***; indirect effect: −0.04, *SE* = 0.03 (*CI*95–0.10, −0.00)	

*N* = 102. MTL = motivation to lead, Bott *SE* = standard error of indirect effect, LL = lower limit, UL = upper limit, corr. 95% *CI* = corrected 95% confidence interval, 5,000 bootstrap replicates; *** = *p* < 0.001 (two-sided); ** = *p* < 0.01 (two-sided); * = *p* < 0.05 (two-sided).

**Figure 5:**
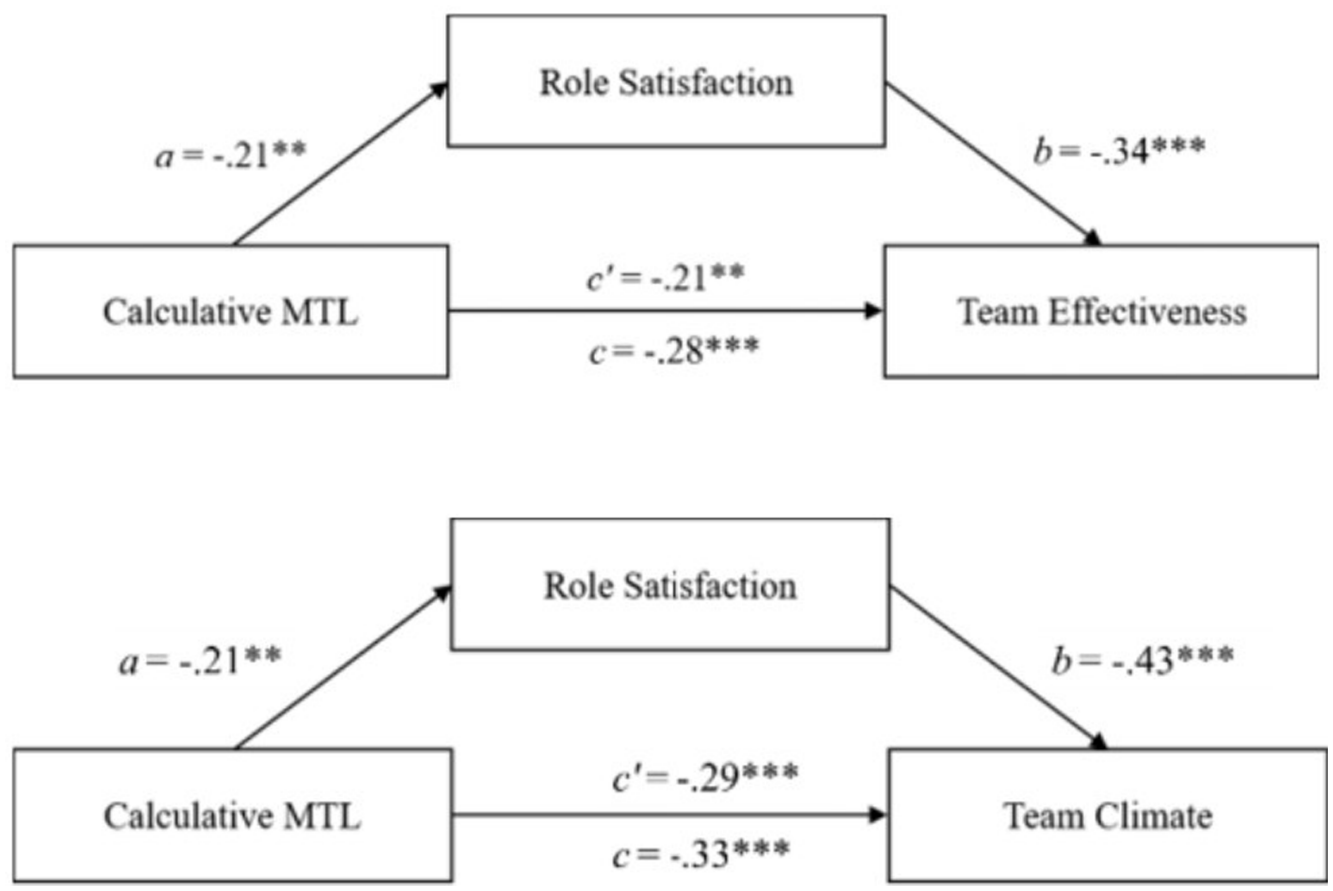
The mediation effects of role satisfaction on the relationship between calculative MTL and team outcomes in the German follower sample. ***p < 0.001, **p < 0.01.

Regarding the relationship between calculative MTL and team effectiveness with the mediating role of role satisfaction, the total effect (*c* = −0.28, *p* < 0.001), direct effect (*c′* = −0.21, *p* = 0.001), and indirect effect (−0.07, 95% *CI* [−0.15; −0.01]) are all negatively significant (see Figure 5). Therefore, a partial mediation is assumed here. The analyses regarding team climate revealed a significant negative total effect (*c* = −0.33, *p* < 0.001) and a significant negative direct effect (*c′* = −0.29, *p* < 0.001) which both were significant. Yet, the indirect effect (0.03, 95% *CI* [−0.10; 0.00]) was not significant (see Figure 5). Hypothesis 4c was confirmed, whereas 4d was rejected.

#### Results for the Chinese sample

For this sample, the same hypotheses as in both German samples were made. Additionally, we predicted positive correlations of normative MTL with team outcomes and role satisfaction and also role satisfaction mediating the positive effects of normative MTL on the team outcomes. Table 5 depicts the means, standard deviations, reliabilities and intercorrelations of the measured scales. We again controlled in our analysis for gender. It again had no effect on the results, so we report results without controls. The Chinese data showed a strong significant positive correlation between affective and normative MTL of 0.59 (*p* < 0.001).

**Table 5:** Means, standard deviations, reliabilities, and correlations of the Chinese employee sample.

Variables	*M*	*SD*	1	2	3	4	5	6	7	8	9	10
1. Gender Participant	1.69	0.48	–									
2. Age Participant	30.68	8.05	−0.11	–								
3. Gender Leader	1.41	0.49	0.39**	−0.17*	–							
4. Age Leader	2.51	0.92	−0.13	0.47**	−0.21**	–						
5. Affective MTL	3.43	0.68	0.02	−0.03	−0.03	−0.05	(0.76)					
6. Calculative MTL	2.71	0.73	0.16*	−0.01	0.07	−0.01	−0.00	(0.79)				
7. Normative MTL	3.33	0.75	−0.01	−0.08	0.06	−0.20*	0.59**	0.09	(0.76)			
8. Role Satisfaction	3.61	0.54	0.17*	0.03	−0.08	−0.04	0.54**	0.19*	0.44**	(0.76)		
9. Team Effectiveness	3.34	0.75	0.12	−0.07	0.22**	−0.05	0.44**	0.06	0.40**	0.28**	(0.91)	
10. Team Climate	3.41	0.80	0.02	−0.18*	0.15	−0.13	0.50**	−0.08	0.47**	0.20*	0.75**	(0.93)

*N* = 164. Gender: 1 = male, 2 = female; Cronbach’s alphas in parentheses; Age Leader: 1 = under 30; 2 = 31–40; 3 = 41–50; 4 = 51–60; 5 = over 60; MTL = motivation to lead; ** = *p* < 0.01 (two-sided); * = *p* < 0.05 (two-sided).

In this data set, we found a positive correlation for affective (*r* = 0.44, *p* < 0.001) and normative (*r* = 0.40, *p* < 0.001) MTL with team effectiveness. Calculative MTL did not correlate with team effectiveness. These results supported Hypotheses 1a and 1c, while 1b was rejected.

For team climate, the hypotheses and the findings were the same. Positive correlations with affective MTL (*r* = 0.50, *p* < 0.001) and normative MTL (*r* = 0.47, *p* < 0.001) and no correlation of team climate with calculative MTL were found, supporting Hypotheses 2a and 2c, while contradicting 2b.

Regarding the third set of hypotheses in the sample, a positive effect was found for affective MTL (*r* = 0.54, *p* < 0.001) and normative MTL (*r* = 0.44, *p* < 0.001). However, a positive significant correlation was also found for calculative MTL (*r* = 0.19, *p* = 0.01). Therefore, Hypotheses 7a and 7c were confirmed, whereas 7b was rejected.

#### Mediation analysis in the Chinese sample

The results of the mediation analysis in the Chinese sample are depicted in Table 6. No significant indirect effect of role satisfaction was found between affective MTL and team effectiveness in the Chinese sample. The same was true for team climate. Therefore, Hypotheses 4a and 4b were not supported.

**Table 6:** Mediation effects of role satisfaction between MTL and team effectiveness and team climate in the Chinese employee sample.

Variables and indices	Role Satisfaction		Team Effectiveness		Team Climate	
	*b*	*SE*	*b*	*SE*	*b*	*SE*
Affective MTL	0.43***	0.05	0.45***	0.09	0.67***	0.10.
Role Satisfaction	--------	--------	0.07	0.12	−0.17	0.12
*R^2^*	0.29		0.19		0.26	
Total, direct and indirect effect	--------		Total effect: 0.48***; direct effect: 0.45***; indirect effect: 0.03, *SE* = 0.05 (*CI*95–0.08, 0.14)		Total effect: 0.59***; direct effect: 0.67***; indirect effect: −0.07, *SE* = 0.06 (*CI*95–0.18, 0.04)	
Calculative MTL	0.15**	0.06	−0.01	0.08	−0.13	0.09
Role Satisfaction	--------	--------	0.39***	0.11	0.32**	0.12
*R^2^*	0.04		0.08		.	
Total, direct and indirect effect	--------		Total effect: 0.05; direct effect: −0.01; indirect effect: 0.06, *SE* = 0.03 (*CI*95 0.01, 0.14)		Total effect: −0.09; direct effect: −0.13; indirect effect: 0.05, *SE* = 0.03 (*CI*95–0.00, 0.12)	
Normative MTL	0.33***	0.05	0.35***	0.08	0.51***	0.08
Role Satisfaction	--------	--------	0.18	0.11	−0.02	0.12
*R^2^*	0.21		.		.	
Total, direct and indirect effect	--------		Total effect: 0.40***; direct effect: 0.35***; indirect effect: 0.06 SE = 0.04 (CI95–0.02 0.14)		Total effect: 0.50***; direct effect: 0.51***; indirect effect: −0.01, SE = 0.04 (CI95–0.09 0.08)	

*N* = 164. MTL = motivation to lead, Bott *SE* = standard error of indirect effect, LL = lower limit, UL = upper limit, corr. 95% CI = corrected 95% confidence interval, 5,000 bootstrap replicates. *** = *p* < 0.001 (two-sided); ** = *p* < 0.01 (two-sided).

Mediation analysis regarding the mediating role of role satisfaction on the relationship between normative MTL and team effectiveness revealed no significant indirect effect. The same is true for the relationship with team climate. Thus, Hypotheses 4e and 4f were both not supported.

**Figure 6:**
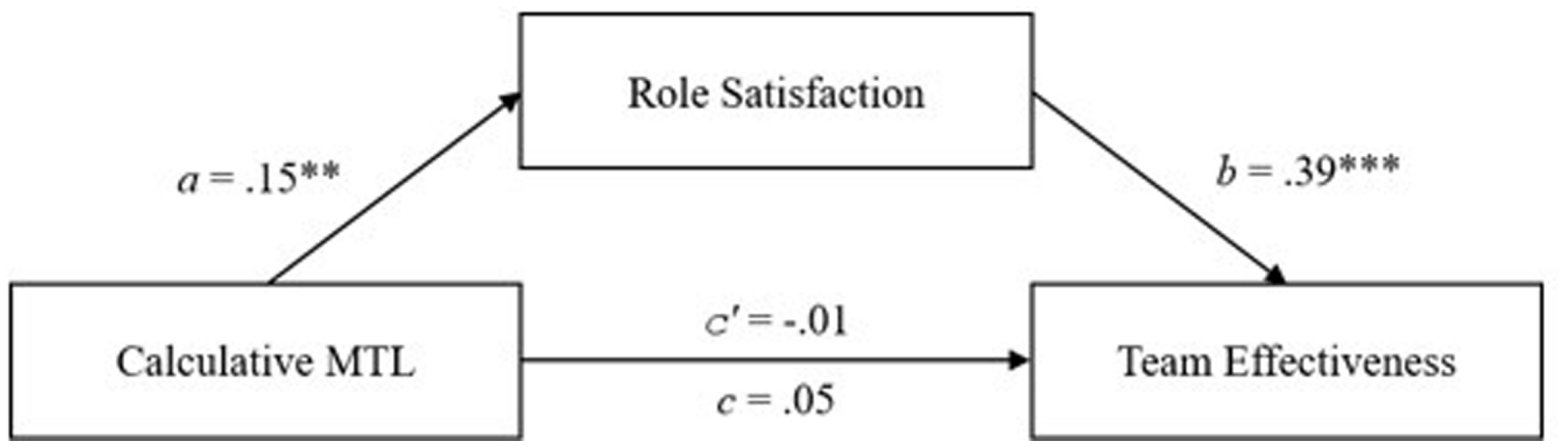
The mediation effects of role satisfaction on the relationship between calculative MTL and team effectiveness in the Chinese follower sample. ***p < 0.001, ** < 0.01.

Regarding the relationship between calculative MTL and team effectiveness mediated by role satisfaction, contrary to Hypothesis 4c, a positive significant indirect effect was found (0.06, 95% *CI* [0.01; 0.14]). Furthermore, a positive significant a-path was found between calculative MTL and role satisfaction (*a* = 0.15, *p* < 0.01), as well as for the b-path between role satisfaction and team effectiveness (*b* = 0.39, *p* < 0.001). The total effect (*c* = 0.05) and the direct effect (*c′* = −0.01) were both not significant (see Figure 6). The indirect effect was significant, therefore, a small partial mediation is assumed.

For the relationship between calculative MTL and team climate, a significant positive indirect effect (0.05, 95% *CI* [0.00; 0.12]) was found, thus rejecting Hypothesis 4d. Yet, neither a significant direct effect (*c′* = −0.13) nor a significant total effect (*c* = −0.09) were found. The a- and b-paths (*a_8_* = 0.15, *p* = 0.01; *b* = 0.32, *p* < 0.01) were both significant.

## Discussion

The goals of this paper were to investigate the differential relationship of affective, calculative and normative MTL on team effectiveness and team climate, to test the influence of perspective (leaders vs. followers), and culture (Germany vs. China) and role satisfaction as a mediator. In the following, we briefly summarize our main findings, outline the theoretical and practical implications and close with implications for future research.

### Summary of main findings

As predicted, affective MTL showed consistent positive relationships with leadership effectiveness (although some of these correlations in the German samples were only marginally or non-significant). The correlations of the extrinsic MTL facets calculative and normative MTL with leadership effectiveness and role satisfaction were not consistent and varied depending on perspective and culture, but not always as predicted. We predicted calculative MTL to have a consistent negative relationship with the leader’s effectiveness and role satisfaction. Surprisingly, these correlations varied strongly between the samples. We found a negative relationship of calculative MTL with leadership effectiveness in the sample with German employees but not in the sample with German managers and also not in in the Chinese employee sample.

For normative MTL we predicted a positive correlation with leader effectiveness, but only for the Chinese sample and indeed, we found this predicted effect in the Chinese sample, showing that following norms is positively perceived in a collectivistic country like China. We did not expect an effect of normative MTL on team effectiveness in the German samples. However, this was only confirmed in the employee sample. For the German leader sample, the correlations were negative, suggesting that normative MTL impairs the leadership quality of German leaders, especially when perceived by the leaders themselves.

Additionally, we introduced the new theoretical construct leader’s role satisfaction, and investigated its effects on the relationship between different MTL facets and team effectiveness and team climate, respectively. As predicted, affective MTL showed a strong positive correlation with role satisfaction among all samples. Normative MTL only correlated positively with role satisfaction in the Chinese sample as predicted. Calculative MTL correlated negatively with role satisfaction in the German samples as predicted, but again in the Chinese sample, calculative MTL showed an unexpected positive correlation, supporting the finding that calculative MTL is seen more positively in China.

Regarding the predicted mediation, we found that a leader’s role satisfaction could indeed explain parts of the relationships between affective and calculative MTL and team effectiveness and team climate, in both German samples. No mediating effects of leader role satisfaction could be found in the Chinese sample.

### Theoretical implications

Overall, our results regarding affective MTL support the notion of SDT that intrinsic motivation is positively associated with satisfaction and performance (Ryan and Deci, 2000), in this case, leadership performance. These findings are independent of culture and the perspective (leader or follower).

In contrast, we found differences in the perception of calculative MTL between German leaders and followers. These differences support the findings of Auvinen et al. (2020), which show that employees give their managers with high calculative MTL lower ratings. Thus, employees seem to see it more critically when managers are motivated by external rewards than managers themselves. In contrast to German followers, Chinese followers seem not to perceive it negatively, when their leaders choose their position for self-serving reasons. This may be explained by the phenomenon that Chinese do not see materialism and collectivism as conflicting. Having higher status and more money reflects well not only on oneself but also on the people associated with oneself such as one’s family or work-team. It is a fulfillment of social expectation and therefore not viewed as a negative aspiration (Awanis et al., 2017). Because the pursuit of higher status and prosperity is seen more as a positive norm in collectivist cultures and is therefore more internalized by people living in collectivistic countries, calculative MTL could also be perceived more strongly as internal, since the distinction between internal and external motivation depends heavily on culture and individual evaluation (Chirkov et al., 2003). This would support the notion that SDT is relevant across cultures, but how the basic needs of SDT are fulfilled depends on context and culture (Chirkov et al., 2003; Ryan and Deci, 2001).

Additionally, it seems to be more important for German leaders than for Chinese leaders to feel comfortable in their leadership role. One explanation could be that the effect of role satisfaction, similar to that of job satisfaction, is lower in collectivistic cultures than in individualistic cultures (Ng et al., 2009; Noordin and Jusoff, 2010). In collectivistic cultures, in strong situations, behavior is driven by situational stimuli, rather than intrinsic beliefs (Snyder and Ickes, 1985). This could mean that high work performance is already expected by the social environment, which might be why leaders already show good work performance even without intrinsic motivation (Ng et al., 2009). Therefore, role satisfaction might not (or only to a limited extend) be applicable to explain MTL effects in collectivistic cultures. Additionally, individual satisfaction might be less important for team outcomes in collectivistic countries than in individualistic countries, because the core concept of collectivism is that the group is more important than the individual, so the satisfaction of the leader should not be as impactful on the team as it is in individualistic countries. The comparably low correlations of role satisfaction with team effectiveness and climate in the Chinese sample are also indicative of this.

The fact that we found differences between the samples does by no means suggest that all Chinese are collectivistic and all Germans are individualistic – there is, of course, variation and overlap, but the results are supporting the idea that this underlying cultural variation influences the general pattern of how leaders and followers react to the different MTL facets.

### Practical implications

This research shows that it is highly relevant for teams and organizations to understand, why leaders are motivated to take on the role of a leader. Normative and especially calculative motivation, when interpreted as external, can impair a leader’s role satisfaction and the outcomes of leadership, and therefore can be detrimental to the team and the organization. This has two main implications for the organization. On the one hand, organizations should ask candidates for leadership positions about their underlying motives. This information might be an important additional basis for the decision-making when leadership positions are to be filled. On the other hand, this finding is relevant for developing an organizational culture. This culture should comprise the mindset that it is not imperative for a career to aspire to leadership positions. On the contrary, employees should be reinsured that they can have a successful career without becoming a leader at some point and that they should aspire for leadership positions only when they are interested in the tasks and the role that comes with them.

However, for societies like China, the implications are less straightforward. In this context, it seems to be no problem if social norms or expectations are a main motivator of pursuing a leadership position, which is on the contrary perceived as highly beneficial. Even pure calculative motivation does not seem to have negative effects but is rather seen positively. This means that in Chinese companies it might be beneficial to support a culture of pursuing leadership positions as a career goal because this perception of fulfilling an organizational norm seems to help the role satisfaction and the effectiveness of leaders. However, this means that not fulfilling this norm might be perceived as even more negative. Thus, organizations with this culture have to develop a good strategy, of how to communicate to employees who are not willing or able to assume a leadership position that they have not failed or broken a norm.

Furthermore, unlike personality, the advantage of MTL is that it can be influenced and developed (Chan and Drasgow, 2001). This means that leadership training and coaching can target MTL. Thus, possible future leaders can talk about and reflect on their leadership motivation. This work on his or her motivation might show whether a change of perspective might be helpful or whether another career path than leadership might be better suited.

### Limitations and future research

All three studies are limited due to the subjective estimation of all variables by only one source (follower or leader) and by gathering a relatively small convenience sample through personal and social networks of the researchers. Future research should conduct studies with leader-follower-dyads in China and Germany to measure the perception of MTL by the leader and the follower and possible effects of (mis)fit. This would also compensate for the fact that no data from a sample of Chinese leaders was collected in this study and this perspective is missing so far. Ideally, it should be a representative sample from a wide range of industries in order to avoid selection bias and to empirically clarify whether the few cases of marginally significant findings were due to low statistical power and thus can be replicated in a larger sample.

Also, both studies used a cross-sectional design which does not allow causal conclusions. Thus, it is also possible that high team effectiveness and good team climate lead to managers being more satisfied with their role as a leader and that they therefore have higher MTL. The problem of causality between performance and satisfaction has been discussed many times in the literature (Judge et al., 2001). Moreover, Chan and Drasgow (2001) suggested that MTL depends on leadership experiences. The quality of these experiences (positive or negative) can presumably influence the strength of MTL. Therefore, it would be interesting to replicate our results in a longitudinal study.

## Conclusion

This research shows the relevance of boundary conditions for the effects of different MTL facets. Specifically, it is important whether the leader’s or followers’ perspective is taken and in which culture leadership takes place. In addition, this study has shown that role satisfaction with the leader role drives some of the effects of MTL, at least in societies like Germany, and that team-related outcomes are also relevant in MTL research.

## Data Availability

The raw data supporting the conclusions of this article will be made available by the authors, without undue reservation.
